# Sea ice directs changes in bowhead whale phenology through the Bering Strait

**DOI:** 10.1186/s40462-023-00374-5

**Published:** 2023-02-07

**Authors:** Angela R. Szesciorka, Kathleen M. Stafford

**Affiliations:** Marine Mammal Institute, Hatfield Marine Science Center, Oregon State University, 2030 SE Marine Science Drive, Newport, OR USA

**Keywords:** Bering Strait, Bowhead whales, Chukchi Sea, Climate change, Migration timing, Phenology, Sea ice, Western Arctic

## Abstract

**Background:**

Climate change is warming the Arctic faster than the rest of the planet. Shifts in whale migration timing have been linked to climate change in temperate and sub-Arctic regions, and evidence suggests Bering–Chukchi–Beaufort (BCB) bowhead whales (*Balaena mysticetus*) might be overwintering in the Canadian Beaufort Sea.

**Methods:**

We used an 11-year timeseries (spanning 2009–2021) of BCB bowhead whale presence in the southern Chukchi Sea (inferred from passive acoustic monitoring) to explore relationships between migration timing and sea ice in the Chukchi and Bering Seas.

**Results:**

Fall southward migration into the Bering Strait was delayed in years with less mean October Chukchi Sea ice area and earlier in years with greater sea ice area (*p* = 0.04, r^2^ = 0.40). Greater mean October–December Bering Sea ice area resulted in longer absences between whales migrating south in the fall and north in the spring (*p* < 0.01, r^2^ = 0.85). A stepwise shift after 2012–2013 shows some whales are remaining in southern Chukchi Sea rather than moving through the Bering Strait and into the northwestern Bering Sea for the winter. Spring northward migration into the southern Chukchi Sea was earlier in years with less mean January–March Chukchi Sea ice area and delayed in years with greater sea ice area (*p* < 0.01, r^2^ = 0.82).

**Conclusions:**

As sea ice continues to decline, northward spring-time migration could shift earlier or more bowhead whales may overwinter at summer feeding grounds. Changes to bowhead whale migration could increase the overlap with ships and impact Indigenous communities that rely on bowhead whales for nutritional and cultural subsistence.

**Supplementary Information:**

The online version contains supplementary material available at 10.1186/s40462-023-00374-5.

## Background

Arctic amplification is changing the climate of the Arctic faster than the global average. Near-surface air temperatures have increased four times faster since 1979 [1] and annual minimum sea ice extent has decreased 13% per decade [2]. Arctic marine mammals, including polar bears (*Ursus maritimus*), seals, walruses (*Odobenus rosmarus*), and whales rely on sea ice for a variety of needs, including access to prey and breeding habitat and for predator avoidance. Increased warming and the continued loss of sea ice will negatively impact some of these ice-obligate species [3–6]. Bowhead whales (*Balaena mysticetus*) are the only baleen whale endemic to the Arctic and rely on phytoplankton blooms tied to annual sea ice melt that feed their zooplankton prey. However, the impacts of a changing Arctic on bowhead whales are less certain than for ice-obligate pinnipeds [3, 5, 7], with some evidence that decreases in sea ice are improving bowhead whale body condition [8].

The distribution and movement of Bering–Chukchi–Beaufort (BCB) bowhead whales in the western Arctic has been established from multiple lines of evidence, including aerial surveys, satellite tagging, passive acoustic monitoring (PAM), and Indigenous knowledge [9–13]. After spending winter in the northwestern Bering Sea, BCB bowhead whales migrate north from April to May through the Bering Strait and into the Chukchi and western Beaufort Seas and then eastward from May to June to the Canadian Beaufort Sea, where they spend the summer feeding. From August to October, they migrate westward toward the Chukotka Peninsula, Russia, before moving south through the Bering Strait, and back into the northwestern Bering Sea for the winter.

Bowhead whale movement is strongly associated with the spatiotemporal availability of prey and distribution of sea ice [14–17]. However, Traditional Knowledge suggests that an increased open water season has shifted the timing of the bowhead spring migration by one month [11, 18, 19]. Additionally, an unknown number of BCB bowhead whales were acoustically documented for the first time spending the entire 2018–2019 winter at what is normally their summer foraging grounds in the Amundsen Gulf and eastern Beaufort Sea [20].

Changes to bowhead whale migration could alter breeding phenology, increase their overlap with human use areas, especially as the Northern Sea Route, Northwest Passage and Transpolar Routes are used increasingly as Pacific-Atlantic shipping routes [21, 22], and impact the ecosystem where they play an important role as a predator [23]. Bowhead whales are one of the most important subsistence species for Native Alaskan and Canadian communities in the Bering, Chukchi, and Beaufort Seas. Thus, changes to the Arctic climate will also affect the people living in the Arctic.

Because of the remoteness and extreme environmental conditions of the Arctic, studying bowhead whale migration over long time periods can be challenging. PAM is non-invasive, allows for long-term, year-round studies of free-ranging whales traveling through or under ice across large geographic ranges, and is a powerful tool for studies of animals that reliably produce sound. From late October to early May, encompassing fall/spring migration through the Bering Strait, bowhead whales produce call sequences repeated as song [24]. Songs can last for hours and are thought to be produced by males as a reproductive strategy [24–26]. During the summer and fall, bowhead whales also produce simple, low frequency (25–1000 Hz) sounds unrelated to song. The sounds are likely used to maintain contact among migrating animals [27], navigate through and under the ice [28, 29], and/or socialize [30]. Because bowhead whales call year-round and the calls are relatively low frequency, they can be detected reliably on hydrophones. The acoustic detections of bowhead vocalizations can be used as an indicator for animal presence, from which migration behavior can be inferred.

Here we use a long-term PAM dataset spanning 2009–2021 to investigate BCB bowhead whale migration through the Bering Strait. Specifically, we examined: (1) the effect of sea ice metrics (i.e., environmental effects) on the timing of the southward migration in the fall from the southern Chukchi Sea through the Bering Strait (hereafter “southward passage”), movement from the Bering Strait into the northwestern Bering Sea in the winter, and northward migration in the spring through the Bering Strait into the southern Chukchi Sea (hereafter “northward passage”); and (2) the temporal trend (i.e., temporal effects) in the timing of the fall southward passage, movement from the Bering Strait to the northwestern Bering Sea in the winter, and northward passage in the spring. We hypothesize that sea ice in the Chukchi Sea is the driving force behind whales migrating through the Bering Strait in the fall and spring and that sea ice in the Bering Strait is the force behind bowhead whales moving out of the Bering Strait and into to the lower latitude Bering Sea/Bering Shelf for the winter.

## Methods

### Data collection and preparation

**Fig. 1:**
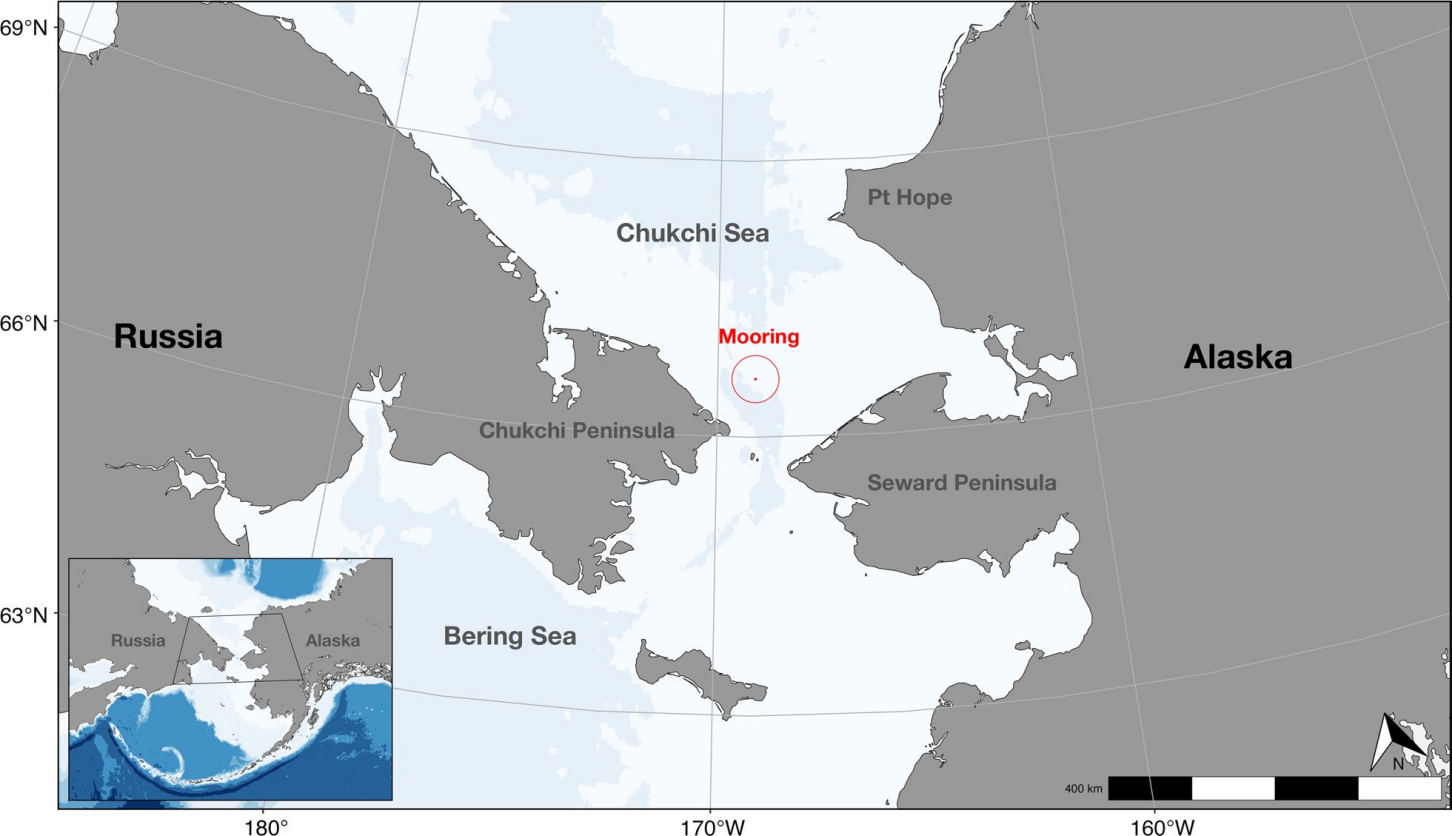
Map of mooring deployment location (~ 66.33° N, 168.5° W; red circle) at the boundary between the Chukchi Sea and the Bering Strait with 30-km detection radius. Black box on inset indicates site within broader global region (i.e., centered between Russia and Alaska). Map made in R [34] with the “ggOceanMaps” (v 1.0.9, [35]) and “ggspatial” (v 1.1.7, [36]) packages

Passive acoustic data were collected by AURAL-M2 (Autonomous Underwater Recorder for Acoustic Listening-Model 2, Multi-Électronique, Inc.) recording packages, which were deployed on moorings (Fig. 1A, Table 1) in the southern Chukchi Sea at the Chukchi Sea/Bering Strait boundary (~ 66.33° N, 168.95° W; Fig. 1B, [31]). The hydrophones sampled at 8192 Hz or 16,384 Hz on duty cycles ranging from 10 to 25 min every hour. The sampling schedule is a trade-off between recording bandwidth, hard drive capacity, and battery pack duration; however, the sample rates are sufficient to capture the entire bandwidth of bowhead whale vocalizations. Each month of acoustic data was converted to a long-term spectral average (LTSA) using Triton software (v 1.93, [32]) in MATLAB (v 2022a, [33]). The LTSAs were averaged across 5 s at 1 Hz resolution.

**Table 1:** Deployment information for 11 migratory seasons (October–May) spanning 2009–2021 including season, sampling rate (Hz), duty cycle (min/h), deployment date (mm/dd/yy format), recovery date, data recording start, data recording end, total acoustic data per deployment (h), water depth (m), and recorder depth (m)

Migratory season	Sample rate (Hz)	Duty cycle (min/h)	Deploy date	Recover date	Data start	Data end	Data (h)	Recorder depth (m)
2009–2010	16,384	12	08/26/09	08/02/10	09/01/09	03/03/10	882	49
2010–2011	16,384	15	08/03/10	07/14/11	08/01/10	02/19/11	1157.5	49
2011–2012	8192	10	07/14/11	07/13/12	10/01/11	05/25/12	956	52
2012–2013	16,384	10	07/13/12	07/04/13	09/01/12	05/17/13	1035	53
2013–2014	8192	20	07/05/13	07/02/14	07/05/13	07/02/14	2822.7	52
2014–2015	8192	20	07/02/14	07/02/15	07/10/14	07/03/15	2840.7	53
2015–2016	8192	22	07/02/15	07/08/16	07/03/15	07/11/16	3294.2	52.5
2017–2018	16,384	18	07/08/17	07/14/17	07/14/17	07/25/18	2710.8	48
2018–2019	16,384	25	08/11/18	09/07/19	08/15/18	04/11/19	1591.7	49
2019–2020	16,384	25	09/07/19	07/09/21	09/10/19	08/31/20	3604.2	49
2020–2021	16,384	25	09/10/20	07/09/21	09/07/20	07/14/21	3108.3	51

All mooring deployment locations were at ~ 66.33° N, 168.5° W

Daily north regional sea ice data for the Bering and Chukchi Seas were extracted from the sea ice index analysis spreadsheets, which are derived from Sea Ice Index V3 [37]. We chose to examine sea ice area (km^2^) as opposed to sea ice extent (km) because even though they are similar, sea ice area is a more quantitative way of describing sea-ice coverage. Sea ice extent is calculated by the number of grid cells in a region with 15% or more sea ice, whereas sea ice area is the total region covered by ice. Because bowhead whales can feed along the sea ice edge and can break through sea ice up to 0.5 m thick with their heads [29], we wanted to use a sea ice metric that would impose greater restrictions on their movement. And although summertime values of sea ice area taken from satellite sensors can be less reliable due to the fact that surface melt appears as open water, we only included sea ice area from October–May, which encompasses the full residence time of bowhead whales migrating south in the fall and north in the spring through the Bering Strait.

### Bowhead whale detection

**Fig. 2:**
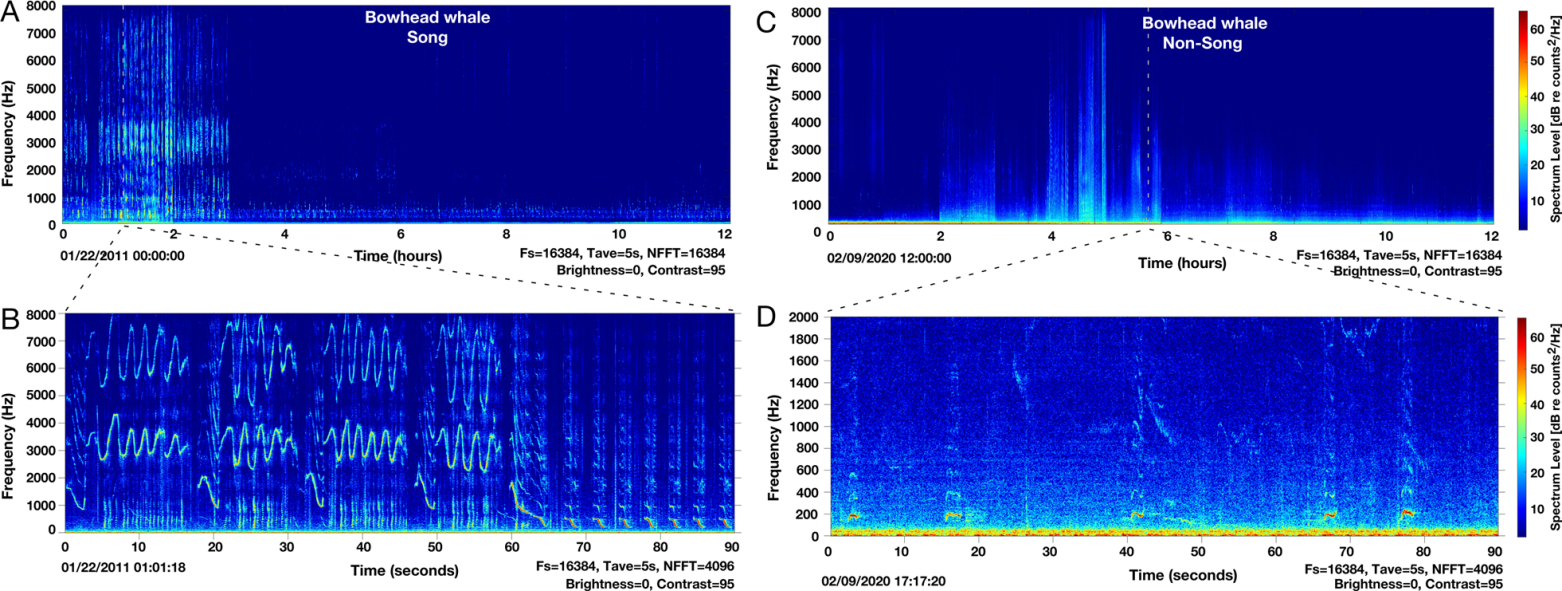
**A** Long-term spectral average (LTSA) with 12 h of acoustic data 22 January 2011. Black dashed lines indicate zoomed-in spectral slice. **B** Zoomed-in spectrogram from LTSA with 90 s of acoustic data depicting bout of bowhead whale song. **C** LTSA with 12 h of calling 9 February 2020. Black dashed lines indicate zoomed-in spectral slice. **D** Zoomed-in spectrogram from LTSA with 90 s of acoustic data depicting non-song vocalizations of bowhead whales. LTSAs plotted with Triton software (v 1.93, [32]) in MATLAB (v 2022a, [33]) (4096 sample FFT, 75% overlap, Hanning window)

Monthly LTSAs spanning October–May were manually visually assessed in Triton for the presence of bowhead whale song and non-song calls (Fig. 2). For each month, an initial 12-h LTSA was scanned. The spectrograms were viewed with a 2048 or 4096 sample FFT, 75% overlap, and Hanning window. The presence of bowhead whale song is typically obvious in a 12-h window (e.g., Fig. 2A). If calls were faint or short, especially for non-song related calls (e.g., Fig. 2C) or to discriminate among overlapping baleen whale species in early fall (e.g., humpback vs bowhead whales), specific hours of interest were assessed at 90 s intervals. Bowhead whale hourly presence was documented for 11 migratory seasons (October–May, which encompass the southward and northern migration of bowhead whales) spanning 2009–2021, excluding 2016 where there were no data available. Hourly acoustic presence was converted to daily and weekly presence by summing number of call hours. To standardize the number of calls by effort, the daily number of call hours were standardized by the proportion of daily effort. In shallow water, similar to that found in the Bering Strait region, most bowhead simple calls should be detectable out to ~ 20 km with the possibility of some signals detectable to a maximum distance of 30 km [38–40].

### Migration timing metrics

Some migratory seasons did not have full data coverage (Table 1); therefore, we could not estimate migration dates based on percentiles around the seasonal distribution of call hours. The onset of the southward passage was defined as the first day with more than 15% of call hours (i.e., day with more than three hours with calls). The cessation of northern migration passage was defined as the last day with more than 15% of call hours. For both southward and northward passage, we explored the first day with any call hours, and 5%, 10%, 20%, and 25% call hours; however, the dates were similar (Additional file 1: Tables S1–S2) and very highly correlated (all *p* ≥ 0.95). We used linear models to assess long-term trends in southward and northward passage dates with the base “stats” package in R (v 4.2.1, [34]). To assess linear relationships between southward and northward passages and sea ice, we tested a number of Chukchi Sea ice area (km^2^) metrics spanning October–May, including monthly values, combinations of months, minimums, and maximums, etc., many of which were significant (Additional file 1: Table S3) and cross-correlated (Additional file 1: Tables S4–S5). We then chose values that balanced level of significance with biological relevance. Significance was set at 0.10 in order to minimize the probability of Type II errors in studies with limited sample sizes [41].

### Bowhead acoustic absence between southward and northward passage

Biologging tag data suggest that bowhead whales migrate through Bering Strait and into the lower latitude Bering Sea for the winter, notably the Anadyr Strait area in the western Bering Sea [13, 42, 43]. To test whether whales were spending more time in the Chukchi Sea and northern Bering Strait than in the northwestern Bering Sea, we quantified the absence between the southward and northward passage into the Bering Strait. We defined this absence as the maximum number of continuous days with less than 15% of call hours (i.e., ≤ 3 h with calls per day). We also explored the number of continuous days with 0 calls and with less than 5% of hours with calls (i.e., < 2 h with calls per day), however, the absences were similar (Additional file 1: Table S6) and highly correlated (all *p* ≥ 0.99). We used linear models to assess the longitudinal trends in number of consecutive gap days with the base “stats” package in R (v 4.2.1, [34]). To assess linear relationships between number of consecutive gap days and sea ice, we used a number of Bering Sea ice area (km^2^) metrics spanning October–May, including monthly values, combinations of months, minimums, and maximums, etc., many of which were significant (Additional file 1: Table S7) and highly cross-correlated (Additional file 1: Table S8). We chose values that balanced level of significance with biological relevance.

## Results

### Bowhead whale calling

Between 2009 and 2021 roughly 22,235 h of bowhead song presence were documented as well as 4669 h of simple song and social/non-song call presence. Because we were interested in the acoustic presence of bowhead whales, if song was detected, we did not then search for calls in the same file. Bowhead whales were predominantly acoustically present from autumn throughout the spring months (November to mid-May) north of Bering Strait in the southern Chukchi Sea. The earliest bowhead whales were detected was 31 October (12 h of non-song presence in 2013) and the latest bowhead whales were detected was 15 May (3 call hours of song and 1 call hour of non-song presence in 2012). Peak months for bowhead whale acoustic presence were December and January, each of which had a daily median of 24 call hours.

### Migration timing and sea ice

**Fig. 3:**
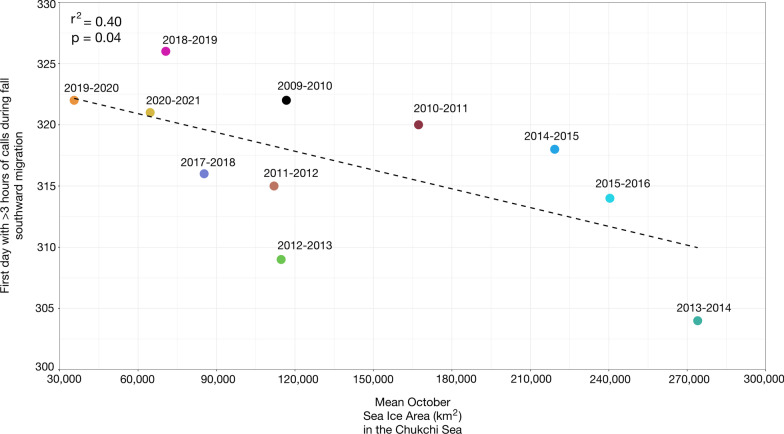
Linear regression (*p* = 0.04, r^2^ = 0.40; y = − 5.11^10^–5^ x + 324) between date of bowhead whale fall southward passage (i.e., first day with > 3 h with calls) through the Bering Strait and mean sea ice area in the Chukchi Sea in October for 11 migratory seasons (October–May) spanning 2009–2021

The southward passage in the fall from the southern Chukchi Sea through the Bering Strait was positively linearly related (*p* = 0.04, r^2^ = 0.40) to mean sea ice area in the Chukchi Sea in October (Fig. 3). Southward passage was delayed in migratory seasons (October–May) with smaller mean sea ice area in the Chukchi Sea in October and earlier in migratory seasons with larger mean sea ice area in the Chukchi Sea in October. There was a 22-day difference in the earliest southward passage and the latest southward passage (Fig. 3). The earliest southward passage (31 October) of bowhead whales from the southern Chukchi Sea and through the Bering Strait occurred during the 2013–2014 season, which was the migratory season with the greatest mean sea ice area in the Chukchi Sea in October (273,984 km^2^). The latest southward passage (22 November) occurred during the 2018–2019 season, which was the migratory season with the third smallest mean sea ice area in the Chukchi Sea in October (70,592 km^2^).

**Fig. 4:**
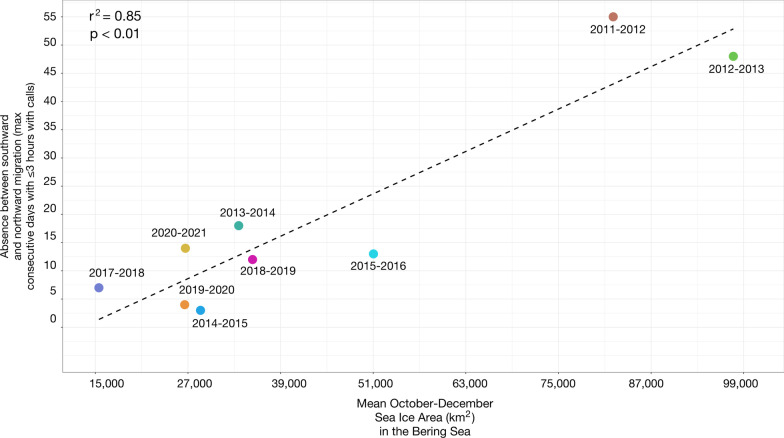
Linear regression (*p* < 0.01, r^2^ = 0.85; y = 6.26^10^–4^ x − 8.29) between the maximum number of consecutive days (~ January–March) with ≤ 3 h with calls per day in the Bering Strait (i.e., absences between southward and northward passage) and mean sea ice area in the Bering Sea in October–December for 9 migratory seasons spanning 2011–2021

The maximum number of consecutive days that whales were acoustically absent between the southward and northward passage was positively linearly related to the mean sea ice area in October-December in the Bering Sea (*p* < 0.01, r^2^ = 0.85) (Fig. 4). Migratory seasons with greater sea ice area resulted in larger absences between whales migrating south in the fall through the Bering Strait and north in the spring. Migratory seasons with less sea ice area resulted in shorter and in some migratory seasons, no absences between whales migrating south in the fall through the Bering Strait and north in the spring. The maximum number of consecutive absences in 2011–2012 (55 days) and in 2012–2013 (48 days) coincided with the top two greatest mean sea ice areas in October-December in the Bering Sea (82,092 km^2^ and 97,656 km^2^, respectively). The maximum number of consecutive absences in 2014–2015 (3 days) and in 2019–2020 (4 days) coincided with the second and fourth smallest mean sea ice areas in October-December in the Bering Sea (28,626 km^2^ and 26,576 km^2^, respectively).

**Fig. 5:**
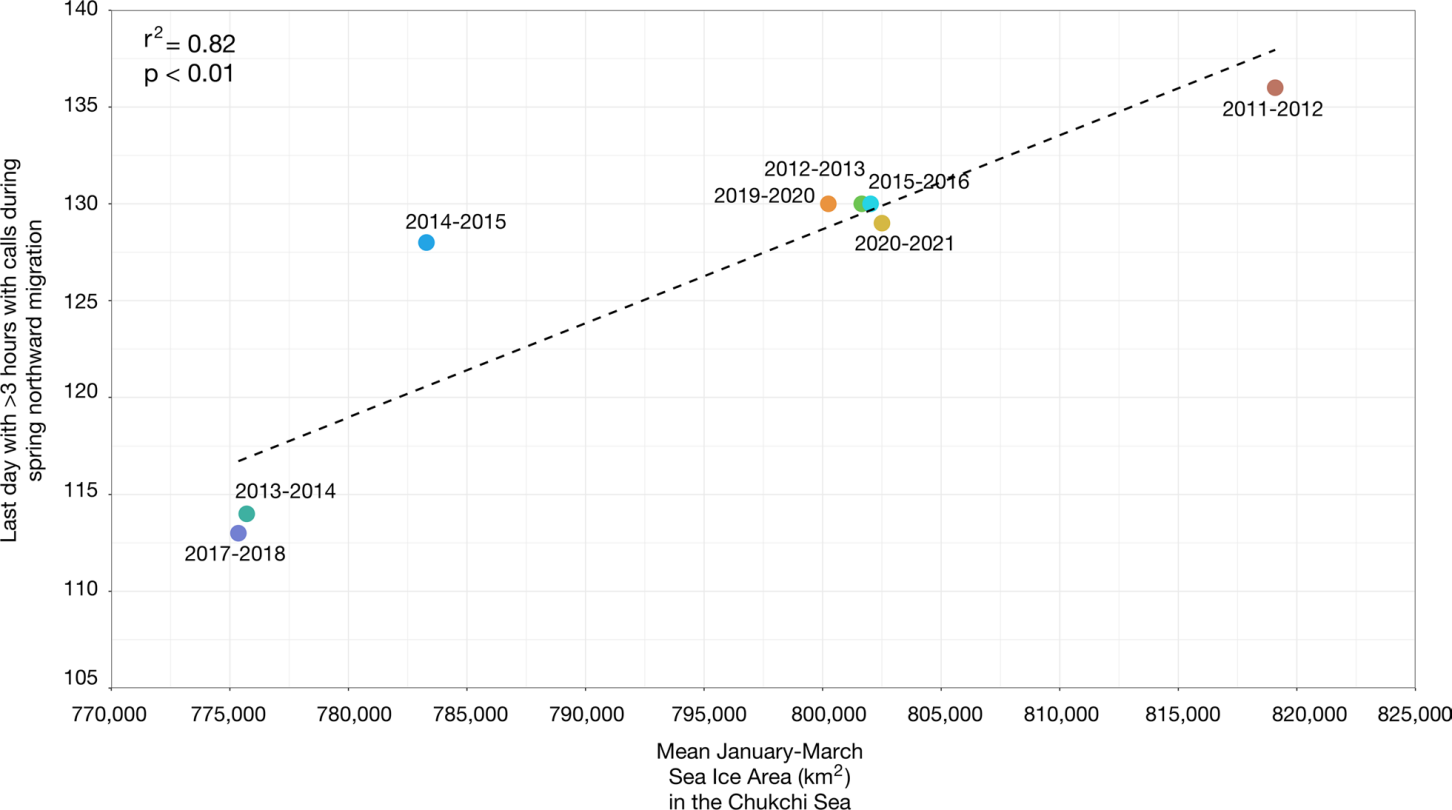
Linear regression (*p* < 0.01, r^2^ = 0.82; y = 4.86^10^–4^ x – 260) between date of bowhead whale spring northward passage (i.e., last day with > 3 h with calls) through the Bering Strait and mean sea ice area in the Chukchi Sea in January–March for 8 migratory seasons spanning 2011–2021

The northward passage in the spring into the southern Chukchi Sea was positively linearly related (p < 0.01, r^2^ = 0.82) to mean sea ice area in the Chukchi Sea in January–March (Fig. 5). Spring-time northward passage was earlier in migratory seasons with less sea ice area in the Chukchi Sea in January–March and delayed in migratory seasons with greater sea ice area in the Chukchi Sea in during these months. There was a 23-day difference in the earliest northward passage and the latest northward passage. The earliest northward passage (23 April) of bowhead whales through the Bering Strait and into the southern Chukchi Seas occurred during the 2017–2018 season (Fig. 5). This coincided with the migratory season with the smallest mean sea ice area in the Chukchi Sea across our study period in January–March (775,360 km^2^) and the smallest November sea ice extent on record. The latest northward passage (May 15) occurred during the 2011–2012 season, which coincided with the migratory season with the greatest mean sea ice area in the Chukchi Sea in January–March (819,087 km^2^).

### Temporal trends

The onset of the southward passage from the southern Chukchi Sea and into the Bering Strait was assessed across 11 migratory seasons between 2009 and 2021, excluding the 2016–2017 season, for which there were no data. Southward passage Julian days (JD) ranged from JD 304 to JD 326 with a median of 318 (95% CI 307–324) (Fig. 3, Table 2). There was no long-term linear trend in the timing of the southward passage from 2009 to 2021 (*p* = 0.43, r^2^ = 0.07). All but two southward passages happened in mid-November between JD 314 and JD 326, a 13-day window. Two early southward passages occurred in the 2012–2013 (JD 309) and 2013–2014 (JD 304) seasons.

**Table 2:** Call metrics for 11 migratory seasons spanning 2009–2021 including arrival Julian day, departure Julian day, and maximum number of consecutive days with gaps between the southward and northward passage

Migratory season	Southward passage Julian day (first day > 3 h with calls)	Northward passage Julian day (last day > 3 h with calls)	N max consecutive gap days (≤ 3 h with calls)
2009–2010	322	nd	nd
2010–2011	320	nd	nd
2011–2012	315	136	55
2012–2013	309	130	48
2013–2014	304	114	18
2014–2015	318	128	3
2015–2016	314	130	13
2017–2018	316	113	7
2018–2019	326	nd	12
2019–2020	322	130	4
2020–2021	321	129	14

nd indicates no data

**Fig. 6:**
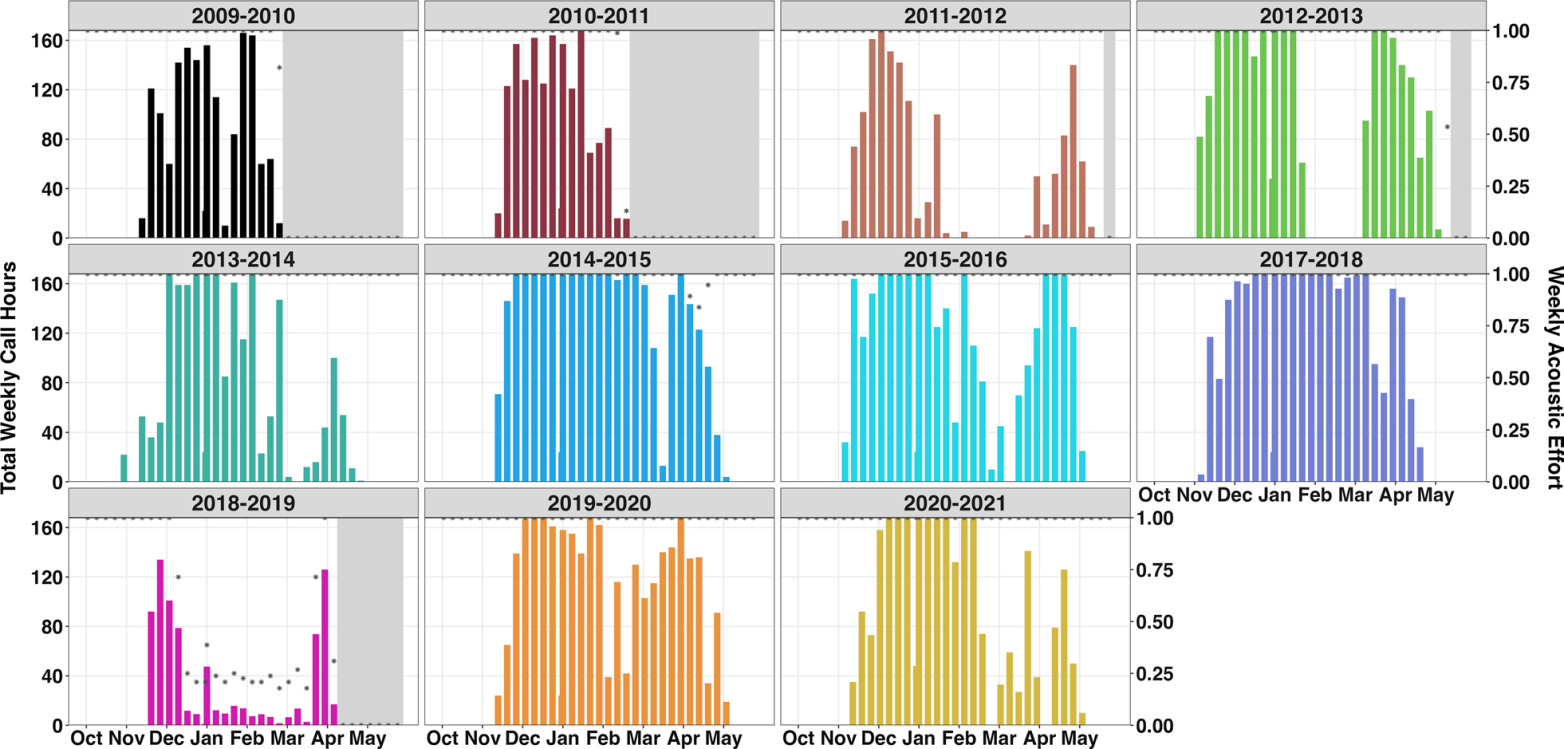
Total weekly call hours of vocalizing bowhead whales from October through May for 11 migratory seasons spanning 2009–2021. Gray bars indicate periods with no acoustic data. Asterisks indicate proportion of weekly effort (secondary y-axis) from the duty cycled data. Most migratory seasons had nearly 100% effort with the exception of 2018–2019. Annual weekly call hour histogram plot made in R [34] with the “ggplot2” package (v 3.3.6, [44]

**Fig. 7:**
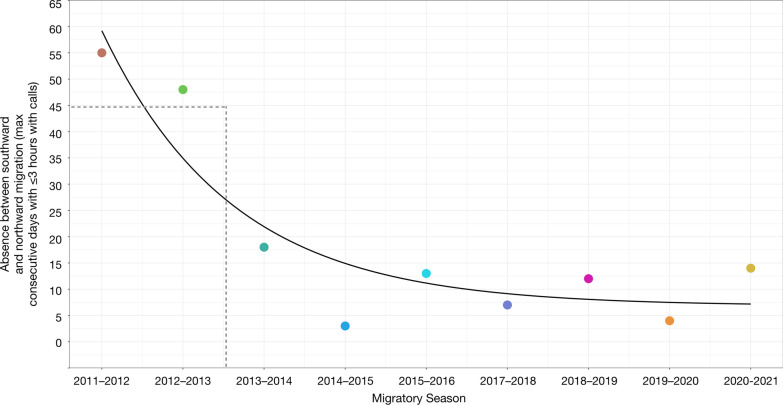
Exponential decay (pseudo r^2^ = 0.85) in absences (i.e., max consecutive days with ≤ 3 h with calls) between whales migrating south in the fall and north in the spring across 9 migratory seasons, driven by the steep decline or stepwise shift (dashed grey lines) after the 2011–2012 and 2012–2013 seasons

The duration of absences from the southern Chukchi Sea between whales migrating south in the fall and north in the spring were assessed across 9 migratory seasons, excluding the 2009–2010, 2010–2011, and 2016–2017 seasons, for which there were no, or incomplete, data. Absences ranged from 3 to 55 consecutive days spanning January–March, with a median of 13 days (95% CI 3–52 days) (Fig. 6, Table 2). Although there was a negatively significant linear trend (*p* = 0.025, r^2^ = 0.54) in the gaps from 2011 to 2021, exponential decay (pseudo r^2^ = 0.85) was a better fit, driven by the steep decline or stepwise shift in number of consecutive days with ≤ 3 h with calls after the 2011–2012 and 2012–2013 seasons (Fig. 7). The 2014–2015, 2017–2018, and 2018–2019 seasons had only three consecutive days with zero call hours and the 2019–2020 season had only two consecutive days with zero call hours.

The timing of the northward passages through the Bering Strait and into the southern Chukchi Sea was assessed across 8 migratory seasons between 2009 and 2021, excluding the 2009–2010, 2010–2011, 2016–2017, and 2018–2019 seasons, where there were no or incomplete data. Northward passage JDs ranged from 113 to 136 with a median of 129 (95% CI 113–134) (Fig. 3, Table 2). There was no long-term linear trend in the timing of the northward passage (*p* = 0.67, r^2^ = 0.03). Most northward passages happened early May, between JD 128 and JD 136, a 9-day window. Two outlier early northward passages occurred in 2013–2014 (JD 114) and 2017–2018 (JD 113).

## Discussion

### The role of sea ice in driving migration timing

We found that both annual southward and northward passages between the southern Chukchi Sea and the Bering Strait were driven by Chukchi Sea ice conditions, while the absence of whales between the southward and northward passages was driven by sea ice in the Bering Sea. This suggests that bowhead whales are forced south into the Bering Strait by Chukchi Sea ice and then moved further south into the Bering Sea by increasing sea ice. The relationship between bowhead whale movement and sea ice is well known [15, 45]. However, our findings highlight the importance of ice on migration timing and patterns, which may be altered drastically by climate change.

While 2018–2019 was a migratory season in our study without good effort coverage (i.e., effort was below 25% for a majority of acoustic files spanning October to May due to instrument issues), sea ice area January–March in the Chukchi Sea in 2018–2019 was close to that of 2017–2018 (782,334 km^2^ and 775,360 km^2^, respectively), which was the migratory season with the earliest end of northern migration (23 April). The linear model predicted a cessation of northward passage as early as 25 April in 2018, providing further evidence of earlier migration north through the Bering Strait and into the southern Chukchi Sea when sea ice is reduced. That same winter, acoustic evidence indicated that some bowhead whales overwintered in Amundsen Gulf and the eastern Beaufort Sea [20]. As sea ice continues to decline, more bowhead whales may overwinter on their feeding grounds.

Although our findings suggest that movement into the Bering Strait and Bering Sea is driven by sea ice, with less sea ice there will be less pressure for whales to migrate as far south in the winter. Tagging studies show that while many bowhead whales migrate to the Bering Sea for the winter, some have remained in the Chukchi Sea and Bering Strait [13, 43, 46]. Additionally, a small number of bowhead whales (~ 500) was documented spending the 2000 and 2001 summers in the Chukchi Sea [47], suggesting flexibility in habitat and diet.

Bowhead whale movement throughout the Arctic is driven by the seasonality of prey hotspots that occur throughout their range [46], similar to other whales whose migration tracks resource waves [48]. Delaying or forgoing winter migration may provide access to more nutrient rich prey, reduce competition from conspecifics or sub-arctic baleen whales, and/or reduce the potential for increased predation pressure from killer whales [49, 50]. However, it is unclear how or if changes to primary and secondary productivity and length of the growing season [51] will outweigh any potential negative impacts from changes in upwelling-favorable winds and zooplankton transport on prey availability [52, 53] and bowhead whale habitat [54, 55].

### Temporal trends

Shifts in whale migration timing have been linked to climate change in temperate and sub-Arctic regions. Fin (*Balaenoptera physalus*) and humpback (*Megaptera novaeangliae*) whales arrived one month earlier in the Gulf of St. Lawrence over a 27-year period [56]; humpback and blue whales (*Balaenoptera musculus*) arrived 120 and 100 days earlier, respectively, in the Gulf of the Farallones over a 24-year period [57]; and blue whales arrived one month earlier to Southern California across a 10-year period [58]. Some Arctic species are also beginning to display migration changes due to climate change. For example, tagged beluga whales (*Delphinapterus leucas*) delayed departure from Beaufort Sea feeding areas in 2004–2012 compared to 1993–2002, coincident with delayed regional sea ice freeze-up in the Beaufort, Chukchi, and Bering Seas [59].

Druckenmiller et al. [55] did not anticipate large changes in the timing of BCB bowhead whale spring migration, though they did predict bowhead whales would spend more time in summer and fall feeding areas, delaying their arrival to wintering areas in the Bering Sea. The cessation of the northward (spring) passages from our study all occurred within a ~ 9-day window across 8 migratory seasons, supporting this hypothesis. However, 8 migratory seasons may not be long enough to identify any significant long-term changes. Additionally, our northward passage metric indicates cessation rather than onset of northern migration, which we were not able to examine with our data.

The onset of the southward (fall) passage from our study occurred within a ~ 2-week window across 11 migratory seasons. The median southward passage (14 November) was not different from averages from tagged bowhead whales in 2008 and 2009 (15 and 26 November, respectively) [42]. However, from 2017 to 2021, the Aleutian Low shifted into the western Bering Sea and the jet stream meandered poleward, supporting southerly winds and reducing ice growth [60]. After 2017, bowhead whale southward passages through the Bering Strait were later than average, suggesting whales may be just starting to delay their arrival to wintering areas in the northwestern Bering Sea. Supporting evidence for this includes bowhead whale fall migration in the western Beaufort Sea occurred consistently later per season from 2008 to 2018, and that timing was influenced by the winter of 2016–2017 during which bowhead whales remained in the Beaufort Sea into mid-January of 2017 [61]. Additionally, bowhead whales were seen and heard in the Canadian Beaufort in the winter of 2018–2019 [20].

Although there was no long-term trend in annual southward or northward passage through the Bering Strait, there was a stepwise shift (i.e., decline) in whale absence from the southern Chukchi Sea during the southward and northward passage across 9 migratory seasons. Chambault et al. [62] predicted northward shifts in Eastern Arctic bowhead whale distribution to cope with climate change. The reduced absence between southward and northern migration through the Bering Strait from our findings supports this, and suggests BCB bowhead whales are shifting northward, potentially overwintering in the southern Chukchi Sea. The first migratory season with no mid-winter gap in acoustic detections coincided with the 2017 Arctic regime shift [60], after which there were very few consecutive days where bowhead whales were absent from the southern Chukchi Sea in the winter of 2018–2019. Taken together, there is an increasing body of evidence that the migration phenology of BCB bowhead whale is changing.

### Future threats for humans and whales

One of the biggest questions is how changes to migration patterns and timing will impact those who rely on bowhead whales for nutritional and cultural subsistence. In response to regional changes over the last 30 years, Yup’ik whalers in the northern Bering Sea have extended their harvest into late fall and winter months [11, 63]. In 2019, Iñupiat whalers in Utqiaġvik, Alaska had not sighted one single bowhead by September and only landed one whale mid-November [64]. Aerial surveys found that the whales had remained unusually far offshore the fall that year [65]. Changes in the timing and distribution of bowhead whales can negatively impact nutritional and cultural subsistence for northern peoples. Thus, predicting future changes to bowhead whale movement and migration timing is of highest priority to local stakeholders.

Continued sea ice decline may increase the overlap of whales with ships, exposure to ship noise, and increase overlap with fisheries and therefore increase entanglement risk. With decreased sea ice, the Northern Sea Route and Northwest Passage are becoming viable Pacific-Atlantic shipping routes where increased vessel traffic will pass through Bering Strait [66]. Increased vessel traffic is likely to increase ship strike injury and death [21, 67]. Modeled bowhead whale habitat use from 2015 to 2017 tag data suggested that they experienced the highest number of underwater noise events and greatest overlap with ship traffic September–October in the southern Chukchi Sea [68], where whales may be spending more time or overwintering in the future. While commercial fishing is currently banned in the high Arctic [69], the Russian Federation has recently started a commercial pollock fishery in the western Chukchi Sea and fisheries research in the region suggests that large biomass of snow crab (*Chionoecetes opilio*) exists in the region [70]. The most recent information on entanglement rates for BCB bowheads, which are over a decade old, suggest that overall, roughly 12% of whales show evidence of entanglement while 50% of large (older) whales show some evidence of entanglement [49, 71]. Should pot and line fisheries move into the southern Chukchi Sea, the potential exists for increases in fishing gear entanglements as has been seen in the northern Bering Sea [72].

## Conclusions

We used a dataset spanning 2009–2021 to examine the long-term trends in the fall southward migration from the southern Chukchi Sea through the Bering Strait, movement from the Bering Strait to the northwestern Bering Sea in the winter, and the spring northward migration through the Bering Strait into the southern Chukchi Sea. The major climate regime shift in 2017 may have driven the apparent step change in the migration patterns and timing of BCB bowhead whales. We hope to use the relationship we found with sea ice to predict future migration timing and compare migration timing at other sites in the western Arctic. Finally, we only looked at presence/absence in terms of number of hours per day with calling, but we do not yet know how many whales were present in the winter in the southern Chukchi Sea and northern Bering Strait. Future visual surveys or satellite images may help quantify the number of whales overwintering in the southern Chukchi Sea.

## Supplementary Information

**Additional file 1.** Supplementary tables with additional information, including migration dates and days absent for each season using different cutoffs; linear regressions between migration dates and days absent with other sea ice area metrics; and cross-correlations among sea ice area metrics.

## Data Availability

The datasets analyzed during the current study are available in the Arctic Data Center, arcticdata.io.

## Abbreviations

BCB: Bering-Chukchi-Beaufort
LTSA: Long-term spectral average
PAM: Passive acoustic monitoring
