# Supplementary material for: Sea ice directs changes in bowhead whale phenology through the Bering Strait
Source: Mov Ecol. 2023 Feb 7;11:8. doi: 10.1186/s40462-023-00374-5 (PMC9903510; doi:10.1186/s40462-023-00374-5)
Supplement: Supplementary file 1 — Additional file 1. Supplementary tables with additional information, including migration dates and days absent for each season using different cutoffs; linear regressions between migration dates and days absent with other sea ice area metrics; and cross-correlations among sea ice area metrics. [file 40462_2023_374_MOESM1_ESM.docx]

**Additional file 1**

**Sea ice directs changes in bowhead whale phenology through the Bering Strait**

**Supplemental Materials**

Angela R. Szesciorka^1*^, Kathleen M. Stafford^1^

^1^Marine Mammal Institute, Hatfield Marine Science Center, Oregon State University, 2030 SE Marine Science Drive, Newport, OR, USA

* corresponding author: angela@szesciorka.com

**Supplementary Tables**

**Supplementary Table 1.** Southward migration (Julian day) each migratory season (2009-2021) using the first day with any call hours and cutoffs at 5%, 10%, 20%, and 25% call hours.

| **Migratory Season** | **Southward any** | **Southward ≥5%** | **Southward ≥10%** | **Southward ≥15%** | **Southward ≥20%** | **Southward ≥25%** |
| --- | --- | --- | --- | --- | --- | --- |
| 2009-2010 | 321 | 322 | 322 | 322 | 322 | 322 |
| 2010-2011 | 320 | 320 | 320 | 320 | 320 | 320 |
| 2011-2012 | 315 | 315 | 315 | 315 | 315 | 315 |
| 2012-2013 | 309 | 309 | 309 | 309 | 310 | 310 |
| 2013-2014 | 304 | 304 | 304 | 304 | 304 | 304 |
| 2014-2015 | 318 | 318 | 318 | 318 | 318 | 318 |
| 2015-2016 | 314 | 314 | 314 | 314 | 314 | 314 |
| 2017-2018 | 309 | 309 | 316 | 316 | 316 | 316 |
| 2018-2019 | 326 | 326 | 326 | 326 | 326 | 326 |
| 2019-2020 | 322 | 322 | 322 | 322 | 322 | 322 |
| 2020-2021 | 321 | 321 | 321 | 321 | 321 | 321 |

**Supplementary Table 2.** Northward migration (Julian day) each migratory season (2009-2021) using the last day with any call hours and cutoffs at 5%, 10%, 20%, and 25% call hours. nd indicates no data.

| **Migratory Season** | **Northward any** | **Northward ≥5%** | **Northward ≥10%** | **Northward ≥15%** | **Northward ≥20%** | **Northward ≥25%** |
| --- | --- | --- | --- | --- | --- | --- |
| 2009-2010 | nd | nd | nd | nd | nd | nd |
| 2010-2011 | nd | nd | nd | nd | nd | nd |
| 2011-2012 | 136 | 136 | 136 | 136 | 133 | 133 |
| 2012-2013 | 130 | 130 | 130 | 130 | 130 | 130 |
| 2013-2014 | 120 | 114 | 114 | 114 | 113 | 111 |
| 2014-2015 | 128 | 128 | 128 | 128 | 123 | 122 |
| 2015-2016 | 130 | 130 | 130 | 130 | 130 | 130 |
| 2017-2018 | 117 | 116 | 114 | 113 | 113 | 113 |
| 2018-2019 | nd | nd | nd | nd | nd | nd |
| 2019-2020 | 130 | 130 | 130 | 130 | 130 | 127 |
| 2020-2021 | 129 | 129 | 129 | 129 | 129 | 126 |

**Supplementary Table 3**. Results of linear regressions between southward and northward passage and Chukchi Sea ice area (km^2^) metrics spanning October through May, which encompasses the fall/spring bowhead whale migration through the Bering Strait.

|  | **Southward Migration** | | **Northward Migration** | |
| --- | --- | --- | --- | --- |
| **Metric** | **p-value** | **r^2^** | **p-value** | **r^2^** |
| Min Oct-May | 0.08 | 0.30 | 0.39 | 0.13 |
| Max Oct-May | 0.16 | 0.21 | 0.02 | 0.65 |
| Mean Oct-May | 0.08 | 0.30 | 0.42 | 0.11 |
| Mean Oct-Dec | 0.05 | 0.36 | 0.85 | 0.01 |
| Mean Jan-Mar | 0.72 | 0.02 | 0.00 | 0.82 |
| Oct Mean | 0.04 | 0.40 | 0.52 | 0.07 |
| Nov Mean | 0.04 | 0.38 | 0.91 | 0.00 |
| Dec Mean | 0.34 | 0.10 | 0.32 | 0.17 |
| Jan Mean | 0.55 | 0.04 | 0.12 | 0.36 |
| Feb Mean | 0.34 | 0.10 | 0.00 | 0.87 |
| Mar Mean | 0.45 | 0.06 | 0.01 | 0.71 |
| Apr Mean | 0.37 | 0.09 | 0.06 | 0.47 |
| May Mean | 0.53 | 0.05 | 0.02 | 0.62 |

**Supplementary Table 4.** Pearson’s cross-correlation values among sea ice area (km^2^) metrics in the Chukchi Sea corresponding to the southward migration each migratory season (2009-2021).

| **Metric** | **Min**  **Oct-May** | **Max**  **Oct-May** | **Mean Oct-May** | **Mean Oct-Dec** | **Mean Jan-Mar** | **Oct Mean** | **Nov Mean** | **Dec Mean** | **Jan Mean** | **Feb Mean** | **Mar Mean** |
| --- | --- | --- | --- | --- | --- | --- | --- | --- | --- | --- | --- |
| Min Oct-May | 1.00 |  |  |  |  |  |  |  |  |  |  |
| Max Oct-May | -0.66 | 1.00 |  |  |  |  |  |  |  |  |  |
| Mean Oct-May | 0.46 | -0.14 | 1.00 |  |  |  |  |  |  |  |  |
| Mean Oct-Dec | 0.57 | -0.31 | 0.95 | 1.00 |  |  |  |  |  |  |  |
| Mean Jan-Mar | -0.28 | 0.60 | 0.39 | 0.10 | 1.00 |  |  |  |  |  |  |
| Oct Mean | 0.88 | -0.63 | 0.62 | 0.75 | -0.27 | 1.00 |  |  |  |  |  |
| Nov Mean | 0.41 | -0.29 | 0.93 | 0.96 | 0.10 | 0.62 | 1.00 |  |  |  |  |
| Dec Mean | 0.30 | 0.06 | 0.90 | 0.89 | 0.40 | 0.43 | 0.85 | 1.00 |  |  |  |
| Jan Mean | -0.20 | 0.35 | 0.15 | 0.06 | 0.59 | -0.29 | 0.01 | 0.40 | 1.00 |  |  |
| Feb Mean | -0.53 | 0.81 | 0.18 | -0.09 | 0.89 | -0.48 | -0.05 | 0.23 | 0.41 | 1.00 |  |
| Mar Mean | 0.16 | 0.12 | 0.52 | 0.28 | 0.69 | 0.19 | 0.27 | 0.27 | -0.03 | 0.50 | 1.00 |
| Apr Mean | 0.23 | 0.08 | 0.78 | 0.56 | 0.67 | 0.25 | 0.58 | 0.59 | 0.10 | 0.50 | 0.84 |
| May Mean | -0.01 | 0.23 | 0.57 | 0.29 | 0.76 | -0.03 | 0.36 | 0.37 | 0.10 | 0.66 | 0.86 |

**Supplementary Table 5.** Pearson’s cross-correlation values among sea ice area (km^2^) metrics in the Chukchi Sea corresponding to the northward migration each migratory season (2011-2021).

| **Metric** | **Min**  **Oct-May** | **Max Oct-May** | **Mean Oct-May** | **Mean Oct-Dec** | **Mean Jan-Mar** | **Oct Mean** | **Nov Mean** | **Dec Mean** | **Jan Mean** | **Feb Mean** | **Mar Mean** |
| --- | --- | --- | --- | --- | --- | --- | --- | --- | --- | --- | --- |
| Min Oct-May | 1.00 |  |  |  |  |  |  |  |  |  |  |
| Max Oct-May | -0.78 | 1.00 |  |  |  |  |  |  |  |  |  |
| Mean Oct-May | 0.41 | -0.07 | 1.00 |  |  |  |  |  |  |  |  |
| Mean Oct-Dec | 0.56 | -0.29 | 0.96 | 1.00 |  |  |  |  |  |  |  |
| Mean Jan-Mar | -0.40 | 0.79 | 0.31 | 0.04 | 1.00 |  |  |  |  |  |  |
| Oct Mean | 0.91 | -0.64 | 0.61 | 0.77 | -0.39 | 1.00 |  |  |  |  |  |
| Nov Mean | 0.38 | -0.24 | 0.93 | 0.96 | 0.03 | 0.62 | 1.00 |  |  |  |  |
| Dec Mean | 0.31 | 0.06 | 0.98 | 0.91 | 0.41 | 0.52 | 0.88 | 1.00 |  |  |  |
| Jan Mean | -0.09 | 0.40 | 0.36 | 0.14 | 0.85 | -0.20 | 0.10 | 0.46 | 1.00 |  |  |
| Feb Mean | -0.63 | 0.93 | 0.13 | -0.13 | 0.90 | -0.55 | -0.08 | 0.22 | 0.56 | 1.00 |  |
| Mar Mean | -0.19 | 0.65 | 0.45 | 0.23 | 0.90 | -0.10 | 0.16 | 0.53 | 0.78 | 0.73 | 1.00 |
| Apr Mean | 0.03 | 0.27 | 0.77 | 0.58 | 0.67 | 0.13 | 0.60 | 0.77 | 0.60 | 0.53 | 0.72 |
| May Mean | -0.36 | 0.56 | 0.46 | 0.20 | 0.84 | -0.33 | 0.29 | 0.49 | 0.70 | 0.80 | 0.69 |

**Supplementary Table 6**. Length of absence between southward and northward passage using the maximum number of days with zero calls, less than 5% of calls, and less than 15% of calls. nd indicates no data.

| **Migratory Season** | **Max consecutive gaps any** | **Max consecutive gaps ≤5%** | **Max consecutive gaps ≤15%** |
| --- | --- | --- | --- |
| 2009-2010 | nd | nd | nd |
| 2010-2011 | nd | nd | nd |
| 2011-2012 | 50 | 50 | 55 |
| 2012-2013 | 45 | 46 | 48 |
| 2013-2014 | 18 | 18 | 18 |
| 2014-2015 | 3 | 3 | 3 |
| 2015-2016 | 13 | 13 | 13 |
| 2017-2018 | 3 | 3 | 7 |
| 2018-2019 | 3 | 12 | 12 |
| 2019-2020 | 2 | 4 | 4 |
| 2020-2021 | 14 | 14 | 14 |

**Supplementary Table 7**. Results of linear regressions between bowhead acoustic absence between southward and northward passage and Bering Sea ice area (km^2^) metrics spanning October through May, which encompasses the fall/spring bowhead whale migration.

|  | **Absence** | |
| --- | --- | --- |
| **Metric** | **P-value** | **R^2^** |
| Min Oct-May | 0.00 | 0.70 |
| Max Oct-May | 0.20 | 0.22 |
| Mean Oct-May | 0.00 | 0.80 |
| Mean Oct-Dec | 0.00 | 0.85 |
| Mean Jan-Mar | 0.00 | 0.77 |
| Oct Area | 0.23 | 0.20 |
| Nov Area | 0.00 | 0.72 |
| Dec Area | 0.00 | 0.84 |
| Jan Area | 0.00 | 0.83 |
| Feb Area | 0.01 | 0.67 |
| Mar Area | 0.01 | 0.68 |
| Apr Area | 0.01 | 0.69 |
| May Area | 0.00 | 0.79 |

**Supplementary Table 8**. Pearson’s cross-correlation values among sea ice area (km^2^) metrics in the Bering Sea corresponding to bowhead acoustic absence between southward and northward passage each migratory season (2011-2021).

| **Metric** | **Min**  **Oct-May** | **Max**  **Oct-May** | **Mean Oct-May** | **Mean Oct-Dec** | **Mean Jan-Mar** | **Oct Mean** | **Nov Mean** | **Dec Mean** | **Jan Mean** | **Feb Mean** | **Mar Mean** |
| --- | --- | --- | --- | --- | --- | --- | --- | --- | --- | --- | --- |
| Min Oct-May | 1.00 |  |  |  |  |  |  |  |  |  |  |
| Max Oct-May | -0.46 | 1.00 |  |  |  |  |  |  |  |  |  |
| Mean Oct-May | -0.41 | 0.97 | 1.00 |  |  |  |  |  |  |  |  |
| Mean Oct-Dec | -0.24 | 0.81 | 0.87 | 1.00 |  |  |  |  |  |  |  |
| Mean Jan-Mar | -0.39 | 0.99 | 0.99 | 0.86 | 1.00 |  |  |  |  |  |  |
| Oct Mean | 0.48 | -0.48 | -0.51 | -0.45 | -0.47 | 1.00 |  |  |  |  |  |
| Nov Mean | -0.34 | 0.83 | 0.83 | 0.91 | 0.83 | -0.56 | 1.00 |  |  |  |  |
| Dec Mean | -0.22 | 0.79 | 0.86 | 1.00 | 0.85 | -0.43 | 0.88 | 1.00 |  |  |  |
| Jan Mean | -0.38 | 0.89 | 0.90 | 0.87 | 0.91 | -0.25 | 0.75 | 0.88 | 1.00 |  |  |
| Feb Mean | -0.29 | 0.98 | 0.98 | 0.84 | 0.99 | -0.48 | 0.84 | 0.82 | 0.86 | 1.00 |  |
| Mar Mean | -0.44 | 0.97 | 0.98 | 0.79 | 0.98 | -0.57 | 0.80 | 0.77 | 0.82 | 0.97 | 1.00 |
| Apr Mean | -0.45 | 0.92 | 0.97 | 0.80 | 0.94 | -0.57 | 0.74 | 0.80 | 0.81 | 0.92 | 0.96 |
| May Mean | -0.45 | 0.94 | 0.97 | 0.79 | 0.96 | -0.48 | 0.77 | 0.78 | 0.87 | 0.94 | 0.94 |
